# The impact of persistent colonization by *Vibrio fischeri* on the metabolome of the host squid *Euprymna scolopes*

**DOI:** 10.1242/jeb.212860

**Published:** 2020-08-28

**Authors:** Eric J. Koch, Silvia Moriano-Gutierrez, Edward G. Ruby, Margaret McFall-Ngai, Manuel Liebeke

**Affiliations:** 1 Kewalo Marine Laboratory, University of Hawaii at Mānoa, Honolulu, HI 96813, USA; 2 Department of Medical Microbiology and Immunology, University of Wisconsin-Madison, Madison, WI 53706, USA; 3 Max Planck Institute for Marine Microbiology, 28359 Bremen, Germany

**Keywords:** Diel rhythm, Hemolymph, Host–microbe, Metabolomics, Squid–vibrio, Symbiosis

## Abstract

Associations between animals and microbes affect not only the immediate tissues where they occur, but also the entire host. Metabolomics, the study of small biomolecules generated during metabolic processes, provides a window into how mutualistic interactions shape host biochemistry. The Hawaiian bobtail squid, *Euprymna scolopes*, is amenable to metabolomic studies of symbiosis because the host can be reared with or without its species-specific symbiont, *Vibrio fischeri*. In addition, unlike many invertebrates, the host squid has a closed circulatory system. This feature allows a direct sampling of the refined collection of metabolites circulating through the body, a focused approach that has been highly successful with mammals. Here, we show that rearing *E. scolopes* without its natural symbiont significantly affected one-quarter of the more than 100 hemolymph metabolites defined by gas chromatography mass spectrometry analysis. Furthermore, as in mammals, which harbor complex consortia of bacterial symbionts, the metabolite signature oscillated on symbiont-driven daily rhythms and was dependent on the sex of the host. Thus, our results provide evidence that the population of even a single symbiont species can influence host hemolymph biochemistry as a function of symbiotic state, host sex and circadian rhythm.

## Abstract (precis)

**Summary:** Study of the squid–vibrio model association reveals that the symbiotic state, the day–night cycle, and the sex of the host together impact the hemolymph metabolome of the host animal.

## INTRODUCTION

Mutualistic interactions between animals and their microbial symbionts are stabilized by the exchange of biomolecules throughout the lifespan of the host. For example, the human intestinal microbiota ferments indigestible polysaccharides into short-chain fatty acids that can be used by the host, while the symbionts receive an energetic benefit from the catabolic process (Bäckhed et al., 2005). Although such host–microbe interactions may occur in a particular tissue location, the molecules generated have far-reaching and lasting effects, e.g. a change in the microbes present in the mammalian gut affects metabolism in the brain and eye (Orešič et al., 2009; Nicholson et al., 2012; De Vadder et al., 2014; Serena et al., 2018). Using a variety of analytical chemistry tools, it is possible to ‘listen in on this concert’ of small biomolecules with metabolomics, providing a better understanding of how microbial metabolism influences host biology (Nicholson et al., 2012; Quinn et al., 2020).

The physiology of a host animal can vary dramatically at several molecular levels, from gene expression to metabolite abundance, and is a natural outcome of aging, circadian rhythm or any perturbance in the microbiota (Schretter et al., 2018). In addition, for sexually dimorphic animals, sex of the host can have a profound effect on metabolism (Klein and Flanagan, 2016; Morgan and Bale, 2017; Barr et al., 2018), as well as host–microbe interactions (Markle et al., 2013; Yurkovetskiy et al., 2013). The activities of the gut microbiota also display circadian patterns that can be sex specific (Kasukawa et al., 2012; Liang et al., 2015). The impact on host physiology due to interacting variables such as developmental stage, circadian rhythms and gender has been long studied. However, the diversity found within consortial associations, such as in the mammalian gut, creates a significant challenge to understanding the role of individual microbial species in the exchange of biomolecules and the chemical signature of the metabolome.

**Fig. 1.**
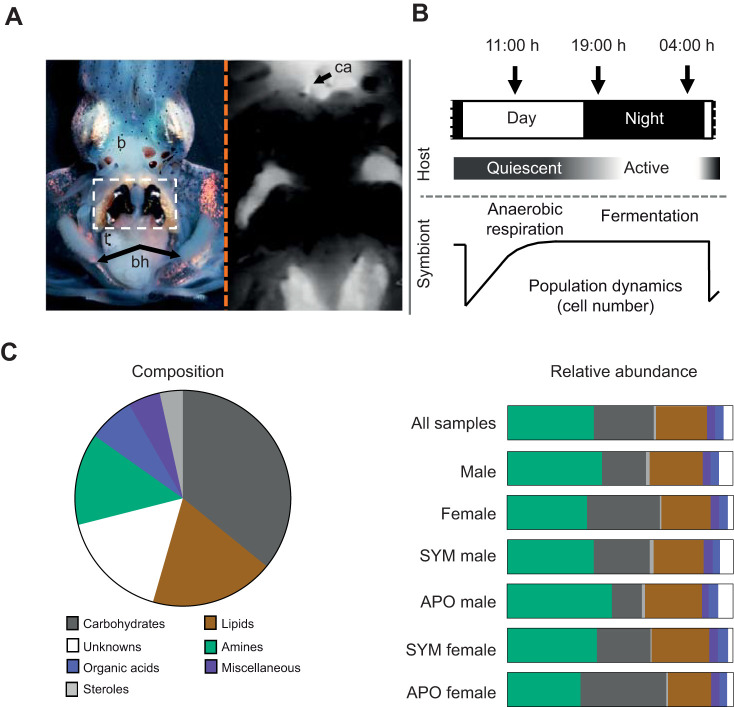
**The squid–vibrio symbiosis and the composite hemolymph metabolome of the host.** (A) Left panel: an adult squid showing the position of the *V. fischeri*-colonized light organ (white box); b, brain; bh, branchial hearts; t, testes; right panel: vascularization in *E. scolopes* (image from Nyholm et al., 2009; with permission from Wiley Publishing)*.* The circulatory system was labeled by injection of CellTracker Orange (Molecular Probes, Eugene, OR, USA) into the cephalic aorta (ca). (B) The daily rhythm of the symbiosis. Numbers at the top represent the times of day that hemolymph was drawn from animals over the day–night cycle; features below illustrate the daily cycles of host activity, as well as symbiont metabolism and population dynamics in the organ. (C) Left panel: the complexity of biochemical composition of the hemolymph under all conditions combined, including male/female and all times of day, was conserved; 120/145 (83%) of metabolites were present in every sample (*N*=33); right panel: representative abundance for each metabolite category; the diagram represents the average total peak areas for each metabolite category as a proportion of the total peak areas within a given host condition (all samples *N*=33, male *N*=16, female *N*=17; SYM male *N*=8, APO male *N*=8, SYM female *N*=8, APO female *N*=9).

The association between the Hawaiian bobtail squid *Euprymna scolopes* and the bioluminescent bacterium *Vibrio fischeri* occurs within a dedicated light-emitting organ which, under natural conditions, is colonized within hours of hatching and matures over several weeks following colonization. This symbiosis presents the opportunity to examine a mutualism that is experimentally tractable. Similar to the symbioses of the mammalian gut, *V. fischeri*: (i) is acquired anew each generation (McFall-Ngai and Ruby, 1991); (ii) resides extracellularly along the apical surfaces of epithelia in dense populations (∼10^11^ cells in the adult squid) throughout the life of the host (McFall-Ngai and Montgomery, 1990); and (iii) affects not only the tissues with which the symbionts directly interact, but also tissues remote from the site of colonization (Moriano-Gutierrez et al., 2019). Another similarity to mammals is that cephalopods have a closed circulatory system that allows efficient transport of biomolecules and immune cells to and from all tissues, including the symbiotic light organ (Nyholm et al., 2009). The well vascularized light organ provides a direct connection between the host circulatory system and the symbiont population (Fig. 1A). As such, the symbiosis is integrated into the overall biological processes of the host throughout its life. In addition to the light organ, female *E. scolopes* have a second symbiotic organ, the accessory nidamental gland (ANG). The ANG harbors a consortium of over a dozen bacterial species that produce bioactive compounds used by the female to protect the squid's eggs from fouling during incubation (Collins et al., 2012; Kerwin and Nyholm, 2018). No successful rearing of female squid without a colonized ANG has been reported (S. Nyholm, personal communication).

Advances in husbandry have enabled the rearing of *E. scolopes* through its entire life cycle (Koch et al., 2014), either symbiotically (SYM) with a desired strain, or aposymbiotically (APO), i.e. in the presence of typical environmental bacteria, but without a sufficient presence of *V. fischeri* to initiate a colonization. Aposymbiotic *E. scolopes* can be maintained indefinitely with no detectable adverse effects on the host; however, in nature, the symbiosis is believed to be obligate, where the light produced by the symbionts provides the host with camouflage from predators (McFall-Ngai and Montgomery, 1990; Jones and Nishiguchi, 2004). Thus, this symbiosis can be studied experimentally at all stages of host maturity, and in any colonization state.

The diel rhythm of the squid–vibrio symbiosis is highly predictable: immediately following initial colonization, a daily pattern of expulsion of ∼95% of the symbiont population occurs each day at dawn (Fig. 1B) (Graf and Ruby, 1998), followed by regrowth from the remaining bacteria in time to promote maximum light emission at night, when the nocturnal host is active (Boettcher et al., 1996). This cycling of symbiont luminescence levels drives a corresponding rhythm in the expression of the host clock gene *escry1* in the light organ (Heath-Heckman et al., 2013). Coincident with the dawn expulsion is a daily shedding of the microvilli of the crypt epithelium (Wier et al., 2010).

In the mature symbiosis, i.e. after ∼4 weeks of colonization, another rhythm is detected wherein, at dawn, and coincident with the effacement of microvilli, symbiont genes indicative of anaerobic respiration of phospholipids are induced; after the host cells repolarize over the following 12 h, the symbiont population changes its metabolism, inducing genes associated with the anaerobic fermentation of the polysaccharide chitin (Wier et al., 2010). The source of the chitin is host hemocytes (Heath-Heckman and McFall-Ngai, 2011), whose nightly trafficking into the light organ increases concomitantly with this change in symbiont metabolism. While the post-dawn anaerobic respiration of host membranes is a pH-neutral process, the fermentation of hemocyte-delivered chitin at night acidifies (i.e. via short-chain fatty acid production) the crypt spaces in a process that is believed to promote luminescence (Schwartzman et al., 2015). In adult *E. scolopes* (Fig. 1A), hemolymph can be withdrawn through the cephalic artery (Nyholm et al., 2009), allowing analysis of both the blood cells (or hemocytes) and of the hemolymph (or cell-free serum). Ready access to the hemolymph provides a unique opportunity to gain insight into the differences in its chemistry in symbiotic and aposymbiotic animals. In addition, it allows an analysis of the changes in hemolymph metabolites due to (i) the day–night metabolic cycle that occurs in the symbiosis (Wier et al., 2010), and (ii) the influence of the sex of the host.

Using the squid–vibrio symbiosis and metabolomic analyses, we explored several ways by which a bacterial population may influence host biochemistry, as well as the magnitude of such effects. Specifically, we compared how the metabolome of the host's hemolymph was influenced by symbiotic state and the profound day–night cycle of the symbiosis, in male and female hosts. The data generated in this study were produced by modulating the presence of a single strain of symbiont, and provide observations that can inform investigations of more complex consortial associations, such as those typical of mammals (McFall-Ngai, 2007).

## MATERIALS AND METHODS

### Colonization procedures

For colonization of the host animal, wild-type *Vibrio fischeri* strain ES114 (Boettcher and Ruby, 1990) was first grown in the nutrient-rich Luria–Bertani salt medium (LBS; Stabb et al., 2001). Prior to inoculation, a subculture was grown to an optical density at 600 nm (OD_600_) of ∼0.6 in seawater–tryptone medium (SWT; Bose et al., 2008). Bacteria were added to the squid's seawater at a final concentration of ∼10^4^ colony-forming units (CFU) per milliliter for ∼12 h. Colonization was confirmed for each animal at 24 h post-inoculation by checking luminescence output using a TD-20/20 luminometer (Turner Design, Sunnyvale, CA, USA).

### Animal husbandry

Specimens of *Euprymna scolopes* Berry 1913 were collected in Maunalua Bay, Hawaii, and transported to the University of Wisconsin-Madison. The squid were maintained in Instant Ocean artificial seawater (Spectrum Brands, Blacksburg, VA, USA) as previously described (McFall-Ngai and Montgomery, 1990). Egg clutches were incubated in 9.5-liter aquaria (Koch et al., 2014). Within 12 h of hatching, juvenile squid were either inoculated with *V. fischeri* or maintained APO overnight, and introduced into rearing vessels the following morning. Hatchlings from different egg clutches were maintained separately. The squid were maintained on a 12 h:12 h light:dark schedule, and reared as previously described for 6 weeks (Koch et al., 2014). Briefly, 25–60 squid were housed per vessel and fed live mysid shrimp (*Mysidopsis bahia*) three to six times per day. At 8 weeks post-hatching, the squid were transferred to 65-liter black high-density polyethylene tanks with 6–10 squid per tank as they approached maturity. Each tank contained a hang-on biofilter to maintain water quality, and 50% (by volume) water changes were performed every 3 days, or more often if required. The squid were fed freshwater shrimp (*Palaemonetes* spp.) overnight with the average size used increasing with that of the squid. Maintenance of SYM or APO conditions in each tank was monitored by periodically plating 50 µl of seawater onto LBS agar, and examining for the presence (SYM) or absence (APO) of colonies of *V. fischeri*.

### Hemolymph sampling

Prior to sampling, squid were confirmed to be either SYM or APO (see above). At least eight animals were sampled for each treatment group (male/symbiotic, male/aposymbiotic, female/symbiotic, female/aposymbiotic) to ensure comparability. Squid were anesthetized with 2% ethanol in seawater, and hemolymph was extracted from the cephalic artery using a 28-gauge needle three times over the day–night cycle: at 11:00 h (5 h after dawn), 19:00 h (2 h after dusk) and 04:00 h (2 h before dawn). Each squid was sampled only once, with 125–200 µl of hemolymph recovered. Following extraction, the hemocytes were removed by centrifuging the samples at 2348 ***g*** for 10 min at 4°C to pellet the cells. Next, 100 µl of the resulting supernatant fraction of the hemolymph was transferred to a 1.5 ml tube, and 50 µl of 0.1 mg ml^−1^ adonitol solution was added as an internal standard. To precipitate proteins, ∼600 µl of methanol at −20°C was added to each sample at a 1:4 v/v ratio of sample/methanol, and the solution was vortexed, incubated at −20°C and centrifuged at 11,363 ***g*** for 30 min at 4°C to pellet precipitated proteins. The supernatant was removed and the protein pellet was resuspended, and the precipitation procedure was performed a second time to ensure maximum extraction of metabolites; the two supernatant fractions were combined and stored at −80°C. Once all samples were obtained, they were dried using a SpeedVac (ThermoSavant, Holbrook, NY, USA), and stored at −80°C.

### Gas chromatography mass spectrometry analysis

Dried hemolymph extracts were derivatized with 40 µl methoxyamine hydrochloride solution (0.02% weight/volume in pyridine) at 37°C for 90 min under constant shaking (1350 r.p.m.). After a 1 min spin-down of the sample, a second derivatization was performed by the addition of 80 µl *N*-methyl-*N*-(trimethylsilyl) trifluoroacetamide (MSTFA) with further heating and shaking at 37°C for 30 min. The derivatized samples were centrifuged for 1 min, and 80 µl of the supernatant were transferred to a small glass vial-inlet for GC-MS analysis.

The GC-MS analysis was performed on an Agilent 7890B gas chromatograph connected to an Agilent 5977A MSD (Agilent Technologies, Santa Clara, CA, USA) as described earlier (Liebeke and Bundy, 2012). Samples were run randomized and injected in split-less mode (1 µl) with an Agilent 7693 autosampler injector and separated using a DB5-MS column (Agilent Technologies) with helium as the carrier gas. After injection, the GC oven-temperature program was started with a temperature of 60°C for 2 min, and the heat increased at 10°C min^−1^ up to 325°C, and held at 325°C for 5 min. Metabolites were assigned using the NIST 2.0 Library (December 2012 version) and, if available, verified by injection of pure standard compounds under the same chromatographic conditions. Metabolites were named ‘unknown’ when MS spectra had a <70% match factor in the NIST Library. Metabolites with a match factor <70%, but which had characteristic mass-spectrometry fragments for a class of metabolites and an approximate retention time window match, were named according to their compound class (e.g. disaccharide or monosaccharide).

### Data analysis

To control for extraction efficiency, all metabolite peak areas were first normalized to the adonitol peak area for each sample. To identify significant metabolites, multivariate and univariate statistical analyses were used sequentially to examine all detected peaks. First, SIMCA-P statistical software (Umetrics, Umeå, Sweden) was used to perform orthogonal partial least squares discriminant analysis (OPLS-DA). The OPLS-DA was used to generate a model that separated two conditions and produced variable importance in the projection (VIP) (Galindo-Prieto et al., 2014). Those metabolites with a VIP>1 were seen as being significant for the separation of two conditions (e.g. SYM *vs* APO). Next, the metabolites with a VIP>1 were further validated using the univariate Wilcoxon rank sum test. All Wilcoxon rank sum tests were performed using Metaboanalyst 4.0 (https://www.metaboanalyst.ca/), with a false discovery rate (FDR) <0.05 considered as significant (Xia et al., 2009; Chong et al., 2018). A hierarchical clustering analysis was performed using Metaboanalyst 4.0, with Euclidean distance measure, and complete linkage for clustering. Table S1 shows data for all metabolites.

## RESULTS

### The *E. scolopes* hemolymph metabolome is complex, and rich in amines and carbohydrates

Sampling body fluids of animals and analysing them with untargeted metabolomic methods allows for discrete determination of metabolic changes over time. In this first study of the host metabolome in the squid–vibrio symbiosis, we chose to focus on the hemolymph to compare features influencing changes in blood chemistry that have been characterized in mammals, specifically influence of symbiosis, daily rhythms and sex of the host. Hemolymph was extracted from both SYM and APO squid at three time points throughout a 24 h cycle (Fig. 1B). From 100 µl of hemolymph per animal, we generated a metabolite profile by GC-MS metabolomics. Untargeted GC-MS analysis of the hemolymph extract revealed a profile of 145 potential metabolites that were classified into seven broad biochemical categories for (Fig. 1C, left, ‘Composition’): carbohydrates (including sugars), lipids, amines (including amino acids), organic acids, miscellaneous and sterols, as well as unknowns, where no sufficient database hit was found (a match factor <70% when comparing the MS spectra with the metabolite library). The four largest biochemical categories by peak number constitute ∼86% of the total metabolome: carbohydrates (36%), lipids (19%), unknowns (17%) and amines (14%) (Fig. 1C, left). All 145 metabolites were present in nearly every hemolymph sample, but the relative abundances varied by sex of the host and its colonization state (Fig. 1C, right, ‘Relative abundance’). The detected metabolite groups in the squid hemolymph are comparable to other animals (i.e. rich in amino acids, sugars and organic acids) (Poynton et al., 2011; Zhang et al., 2013; Deng et al., 2015).

By taking peak area as an approximation of relative abundance, amines were found to be the most highly represented metabolite category, with an average of 38% of total peak areas in the hemolymph; however, they accounted for only 14% of the total number of peaks (i.e. Fig. 1C, left, ‘Composition’). In contrast, both the unknowns and the carbohydrates decreased when comparing total number of peaks with total peak areas, respectively (Fig. 1C, right). These data indicate that amines, e.g. valine and leucine, occur in high concentrations, while carbohydrates, e.g. fucose and trehalose, are in low concentrations in the hemolymph relative to the other metabolites. The majority of unidentified metabolites showed small peaks in the hemolymph metabolome; only unknown sugar #6 was an average-sized peak.

The category distribution in the hemolymph metabolomes was generally similar between the sexes. The largest differences were contained in the carbohydrate and amine categories. Both of these categories represent major hemolymph metabolite groups, with 32% for carbohydrates and 35% for amines in females, whereas males showed proportionally less carbohydrate and more amine (20 and 42%, respectively). When further separated by colonization state, male hemolymph was most affected in these two major metabolite groups (Fig. 1C, right). Colonized males exhibited a higher relative abundance of carbohydrates, but a decreased relative abundance of amines. In contrast to the males, the relative abundance of carbohydrates in female hemolymph decreased with colonization, while that of the amines increased. In addition, the relative abundance of the lipids category was increased in colonized females (Fig. 1C, right). These results show that the hemolymph contains a general metabolite pool that is constitutively produced by the host. However, the factors of host sex and colonization state of the light organ clearly affect the abundances of metabolites in the hemolymph, especially the major categories of carbohydrates and amines.

### The hemolymph of SYM males has increased abundance of most metabolites

**Fig. 2.**
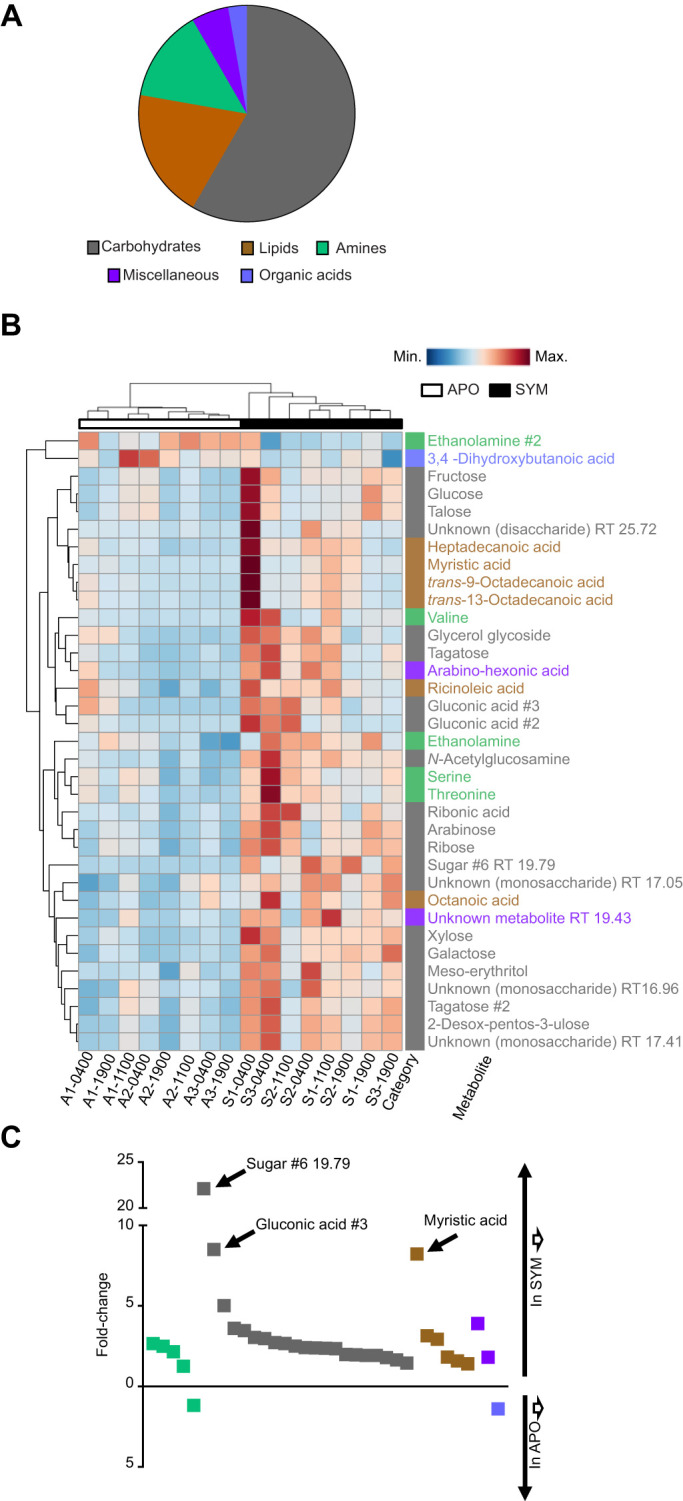
**Metabolites in male hemolymph significantly affected by symbiont colonization.** (A) Biochemical composition of the 35 metabolites that were significantly affected by symbiosis. (B) A heatmap showing unsupervised clustering of the relative abundances of the 35 significant metabolites. The retention times (RT) of the unidentified compounds are given. (C) The fold-change in relative abundance of differentially regulated metabolites under SYM and APO conditions. The metabolites are ordered from the highest to the lowest fold-change within each category. Metabolites increased in SYM are above the *x*-axis, and metabolites increased in APO are below the *x*-axis. SYM male *N*=8, APO male *N*=8.

We chose to examine the effect of colonization on individual metabolite levels only in males, because their sole high-density, persistent symbiosis is the binary light organ association with *V. fischeri* (i.e. they have no ANG). The light organ of *E. scolopes* is well vascularized, creating a direct connection between the symbiont-containing tissues and the circulatory system (Fig. 1A). Comparison of all 145 detected metabolites in male SYM and APO hemolymph across all time points resulted in 35 metabolites (24%) from five categories significantly affected (VIP>1 and FDR<0.05; see Materials and Methods) by colonization. Within the significant metabolites, the most highly represented biochemical categories were carbohydrates with 21 metabolites (60%), followed by lipids with six metabolites (17%). Also affected were five amines (14%), two miscellaneous metabolites (6%) and one organic acid (3,4-dihydroxybutanoic acid; 3%) (Fig. 2A).

Of the metabolites significantly affected by symbiosis, all but two were increased under SYM conditions relative to APO (Fig. 2B,C). Ethanolamine and 3,4-dihydroxybutanoic acid were the only metabolites to be significantly lower in the SYM hemolymph. Although the majority of significantly different metabolites were increased between 1.5- and 5-fold in SYM hosts, three metabolites exhibited higher differences. The putative carbohydrates, sugar #6 and gluconic acid #3 were increased under SYM conditions 22- and 8.5-fold, respectively. In addition, the fatty acid myristic acid was increased 8.2-fold in SYM hemolymph relative to that in the APO state (Fig. 2C).

With the exception of sugar #6, which was absent or near the limit of detection in the APO hemolymph, all 35 of the metabolites significantly affected by colonization were present under both SYM and APO conditions. Furthermore, all metabolites but ethanolamine and 3,4-dihydroxybutanoic acid were increased in SYM relative to APO. Therefore, colonization of the light organ results in an increased abundance of hemolymph metabolites, either directly from the symbiont or through induced production by the host.

### Colonization affects the hemolymph metabolome over the diel cycle

Previous transcriptomic and imaging studies have shown that the squid–vibrio symbiosis expresses several diel rhythms (Schwartzman et al., 2015; Wier et al., 2010). To explore more deeply the role that the daily symbiotic rhythm plays in shaping the host metabolome, we compared SYM and APO hemolymph from males at three different time points across a diel cycle: day (11:00 h) dusk (19:00 h) and night (04:00 h) (Fig. 1B).

Hierarchical clustering analysis grouped the individual hemolymph samples by similarity in their metabolite abundances. The analysis revealed that the SYM hemolymph metabolome is different from the APO metabolome as samples clustered apart from each other (Fig. 2B). Under SYM conditions, the hemolymph metabolomes were mainly grouped by time points, in the hierarchy of 04:00 h next to 11:00 h, and 19:00 h samples (Fig. 2B). In contrast, the sample clustering analysis showed that the metabolome of APO males for the most part did not group together coherently according to the time of sampling. The difference in clustering between SYM and APO indicates that the presence of the symbionts is altering the hemolymph metabolome in a time-dependent manner over the diel cycle. Across all 145 detected metabolites, on average we observed a lower coefficient of variation (CV) per metabolite under SYM conditions compared with APO samples. When comparing SYM and APO over the different time points, the largest difference in average CV occurred at 04:00 h (CV_SYM_=44.4 and CV_APO_=58.3 at 04:00 h) (Fig. S1). These results indicate that SYM hemolymph showed more stable metabolite patterns over the diel cycle, while the absence of symbionts seemed to increase stochasticity (Fig. S1).

**Fig. 3.**
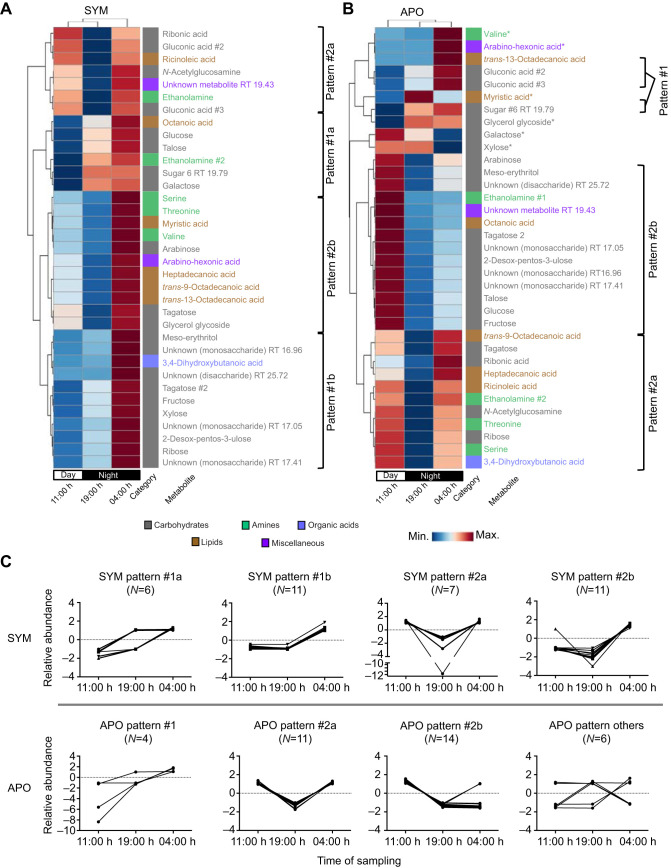
**Colonization affects the diel rhythm of the metabolome.** (A,B) Hierarchical clustering analysis of the 35 metabolites with significantly different abundances, averaged by time of sampling under SYM (A) and APO (B) conditions. The biochemical category is displayed to the right of each metabolite. Metabolites that followed a common abundance pattern are grouped together (C); the six metabolites that had other patterns are indicated by asterisks in B. SYM male *N*=8, APO male *N*=8.

To identify general diel rhythms in the hemolymph metabolites, we next performed hierarchical clustering in which the average metabolite concentrations per time point were compared across the diel cycle (Fig. 3). The overall pattern in SYM animals was a general up-regulation relative to the other time points just before dawn, with 33 (94%) of the metabolites having their highest concentrations at 04:00 h (Fig. 3A). The hemolymph metabolites in SYM animals showed separation into two major patterns over the day–night cycle that were further separated into two subgroups each (#1a, #1b and #2a, #2b). The metabolite levels were generally highest at 04:00 h, with 11:00 h and 19:00 h having either low or moderate levels. The majority of carbohydrates (14/21, 67%) exhibited a general increase throughout the day (#1a and #1b; see Fig. 3C), whereas metabolites following both #2a and #2b showed lower levels at 19:00 h.

In APO males, hemolymph metabolites displayed different patterning over the diel cycle relative to SYM (Fig. 3B). Similar to SYM, the APO metabolites were grouped into two general patterns (patterns #1 and #2). There was a general up-regulation at 11:00 h, with 22 metabolites (63%) showing their highest concentration at this time point. Pattern #2 subgroups (#2a and #2b) showed 19:00 h consistently having low concentrations, while 11:00 h and 04:00 h displayed low, medium and high concentrations. In both SYM and APO, pattern #1 metabolites were mainly represented by sugars (Fig. 3). Six APO metabolites could not be grouped into a pattern as they did not share a trend with at least two other metabolites, indicating that a greater variance existed under APO conditions. These results suggest that the absence of *V. fischeri* perturbs the daily rhythms of the hemolymph metabolome.

### Colonization affects the hemolymph metabolome in both sexually dimorphic and sex-independent manners

**Fig. 4.**
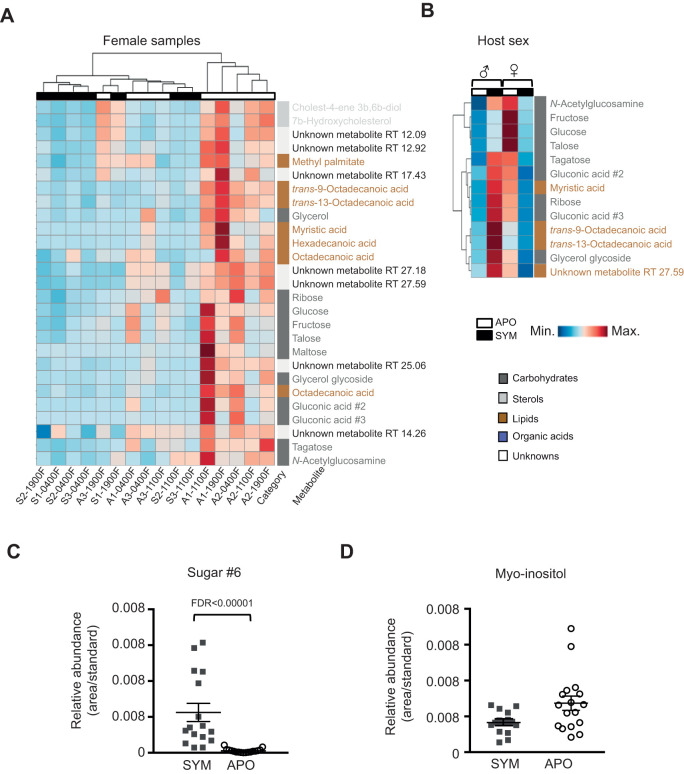
**The interaction of the sex of the host and** **colonization on the metabolome.** (A) Heatmap showing unsupervised clustering of the 27 significantly different metabolites between SYM and APO females. (B) Heatmap displaying clustering analysis of the relative abundance of the 13 metabolites that were significantly differently regulated by colonization in both males and females. (C) The presence of the metabolite ‘sugar #6’ is significantly increased under SYM conditions in both males and females. (D) The metabolite myo-inositol is decreased, although not significantly when correcting for multiple comparisons, under SYM conditions in both males and females. A univariate Wilcoxon rank sum test was used; mean values with s.e.m. are shown. SYM male *N*=8, APO male *N*=8, SYM female *N*=8, APO female *N*=9.

To determine whether the sex of the host affects the impact of colonization on the metabolome, we compared SYM and APO females and found 27 significantly different metabolites (VIP>1 and FDR<0.05) (Fig. 4A). These metabolites consisted of 14 carbohydrates (52%), seven lipids (27%), three unknowns (11%), two sterols (7%) and one organic acid (4%). Unlike in males (Fig. 2), the amines were not significantly affected by colonization in females. All 27 of the significantly different metabolites were present under both SYM and APO conditions. However, in contrast to males, these 27 significant metabolites were all decreased in SYM relative to APO females (males had 34/35 significant metabolites increased in SYM/APO). A total of 13 metabolites (nine carbohydrates and four lipids) were significantly different between SYM and APO conditions for both males and females (Fig. 4B), indicating that these molecules are affected by symbiosis in both sexes. All 13 of these metabolites displayed sexual dimorphism, i.e. relative to APO, each was significantly increased under SYM conditions in males, while significantly decreased under SYM conditions in females.

To investigate further the effects of colonization on the metabolome in a sex-independent way, the male and female data were combined across all three time points. When comparing all SYM to all APO samples, the partially characterized sugar #6 was determined to be significantly affected by colonization (FDR<0.05), i.e. increased in SYM relative to APO for both males and females (Fig. 4C). While sugar #6 was included in the 35 significant metabolites for males, it did not meet our significance cut-off in females (VIP<1), possibly due to being present at low abundance. The other 144 metabolites were not significantly different between SYM and APO when the male and female metabolome datasets were combined, making sugar #6 a candidate marker for colonization by *V. fischeri*. The metabolite myo-inositol, while not significantly different between SYM and APO, when correcting for multiple comparisons (FDR>0.05) exhibited down-regulation in SYM for both males and females (Fig. 4D). These results indicate that while the majority of the metabolome exhibits sexual dimorphism, some metabolites are affected by colonization independently of host sex.

## DISCUSSION

In this study, we showed how persistent colonization of the light organ dramatically alters the *E. scolopes* hemolymph metabolome. The squid–vibrio symbiosis provides the opportunity to study host development from embryogenesis through to full maturity, both in the presence and absence of its symbiont, while maintaining the host's typical exposure to other environmental microbes. Taking advantage of these properties, the metabolomics data here showed that the absence of *V. fischeri* in the light organ significantly affects ∼25% of the hemolymph metabolites of adult male *E. scolopes*. By sampling three times over a 24 h diel cycle, we showed that the levels of these biomolecules follow rhythms, with the presence of the symbiont being a key determinant of the patterns. Also, performing these experiments in both male and female squid allowed us to show that the effects of colonization on the metabolome can be further affected by the sex of the host. These results indicate that the presence of a single species of symbiont can modulate the daily rhythms of small molecules circulating throughout its host.

It should be noted here that the aim of this study was not to derive a comprehensive view of all circulating metabolites, but rather to focus on the influence of symbiosis on the metabolome. For example, we only considered those amino acids that were specifically affected by the symbiotic state; others were detected, but not significantly altered (e.g. valine, leucine, proline, phenylalanine and others). Furthermore, this study focused on blood metabolome, and did not cover the topic of tissue metabolome, which will be an interesting topic of future studies.

Interactions between animals and bacteria rely upon an exchange of a variety of biochemical products, such as metabolites. Using metabolomics, the mammalian gut symbiosis has been studied extensively, helping to define how a consortium of microbes can affect host biochemistry (Nicholson et al., 2005). One important discovery from our study is that, under both SYM and APO conditions, the hemolymph metabolome consisted of the same 145 metabolites, with the exception of a single sugar (‘sugar #6’) detected only in the SYM. The presence of the other 144 metabolites in APO hemolymph indicates that the host likely produces these metabolites. In contrast, a rich array of symbiosis-specific metabolites can be identified in mice, compounds that are completely absent in the germ-free condition (Li et al., 2018; Matsumoto et al., 2018). However, a key difference is that mammals derive a nutritional service from their gut microbes, e.g. the breakdown of plant polysaccharides, and provision of short-chain fatty acids and other fermentation products (Bäckhed et al., 2005). In contrast, there is no evidence that the squid host benefits metabolically from the colonization of the light organ. Studies with insect symbioses in which the host also provides the raw nutrients, but receives specific symbiont metabolites as a benefit, have shown both general effects on metabolites related to energy production and more specific effects (e.g. essential amino acid production) (Wang et al., 2010; Zheng et al., 2017).

The easy detection of sugar #6 in all SYM samples, but its apparent absence in APO, provides evidence that either the host produces that metabolite only when colonized by the symbiont or that the symbiont is the source of this metabolite, which has diffused from the symbiont population into the hemolymph. Unfortunately, sugar #6 could only be partially characterized as a sugar derivative, i.e. the closest matches in the metabolite database originated from sugars. Analysis of secreted metabolites from *V. fischeri* grown in minimal media culture did not detect sugar #6, indicating that, if it is a product of symbiont metabolism, the sugar is not produced by the bacteria under standard culture conditions (data not shown). Whether the metabolite is produced directly by *V. fischeri* within the light organ, or is produced by the host in response to the symbiont, remains to be determined.

A possible reason for the detection of only one colonization-dependent metabolite is the use of GC-MS as our analytical tool. While this method reveals the presence of many hemolymph metabolites, in particular carbohydrates and amino acids, only a few metabolites from other classes (e.g. lipids) can be detected. Several different analysis platforms are needed to reveal the full range of constituents present in hemolymph, especially classes consisting of diverse metabolites such as lipids (Psychogios et al., 2011). To assess whether our method would detect metabolites typically present in cephalopod body fluids (Deffner, 1961; Storey et al., 1979), we subjected to our GC-MS assay synthetic standards of two such chemicals: isethionic acid and octopine. Isethionic acid could be derivatized and detected by our method; however, no such peak was observed in any samples of host hemolymph. In contrast, we could not detect synthetic octopine following a derivatization step; thus, although the expression of host transcripts encoding octopine dehydrogenase (Weir et al., 2010) is suggestive that octopine might be present, our GC-MS assay would be unable to detect it in the hemolymph samples. Further studies, applying additional analytical methods, such as liquid chromatography mass spectrometry (LC-MS), would provide a more complete description of the hemolymph metabolome, and may lead to the identification of molecules specifically produced by the symbiont.

What is clear from this study is that colonization resulted in widespread alterations in the hemolymph metabolome. These changes occur principally in metabolite abundance, and could be either direct (i.e. production or consumption by the symbiont) or indirect (i.e. altering host production or consumption). In male squid, ∼25% of all hemolymph metabolites were significantly and reproducibly different between SYM and APO animals. Similarly, a mass spectrometry-based metabolomics study comparing germ-free to conventionalized mice showed that the abundances of ∼10% of metabolites changed significantly between the two conditions, with the majority being increased under the SYM condition (Wikoff et al., 2009). The differences we observed in the squid metabolite signature in APO could arise from either an alteration in developmental maturation when *V. fischeri* was missing, or the absence of an expected metabolic demand by the symbiont population.

By sampling at three time points throughout a 24 h cycle, we observed that colonization of the light organ coincided with a diel variation of metabolite abundance. The 35 metabolites significantly different in males due to colonization could be grouped into two temporal abundance patterns in SYM animals, whereas several of these metabolites did not vary in the same temporal pattern in APO. Specifically, in APO hemolymph, 29 of the significant metabolites grouped into the same two patterns as seen in SYM; however, six of the metabolites did not group into a pattern. This greater likelihood of patterning in SYM indicates that the presence of *V. fischeri* resulted in more constrained metabolic rhythms. In an analogous manner, the microbiota of vertebrates has been reported to influence rhythms in the host metabolome directly (Thaiss et al., 2016; Weger et al., 2019).

The pattern of glucose abundance in squid hemolymph throughout the day was different between SYM and APO (Fig. 3). During the day (11:00 h) in SYM, and at all three time points in APO, average glucose levels were between 95 and 200 µg ml^–1^, similar to what has been reported in other cephalopod hemolymph sampled at unspecified times of day (Storey et al., 1979; Lamarre et al., 2012; Linares et al., 2015). However, at night (19:00 h and 04:00 h), SYM animals exhibited significantly elevated glucose levels, with average concentrations of approximately 300 and 400 µg ml^–1^, respectively. In contrast, dimethylglycine, an abundant metabolite by average peak area, exhibited the same daily pattern for both SYM and APO hemolymph, with highest abundance at 19:00 h. While dimethylglycine levels were not statistically significantly different overall when comparing SYM with APO, this metabolite was, on average, decreased in SYM relative to APO at all three time points (Fig. S2). Taken together, these results suggest that some aspects of host physiology (e.g. glucose metabolism) may be altered by *V. fischeri* in a manner associated with time of day, while in others symbiosis has no influence on their daily pattern of regulation.

The SYM and APO metabolomes also exhibited different periods of highest abundance over the three times of sampling. In SYM, the 35 significant metabolites were most abundant at 04:00 h, while in APO they were most abundant at 11:00 h. This difference may be relevant to the daily rhythm of the squid–vibrio symbiosis, which is ‘reset’ every day at dawn, when ∼95% of the crypt contents (i.e. the symbionts and the matrix in which they are embedded) are vented into the surrounding seawater (Boettcher et al., 1996; Graf and Ruby, 1998). Because the majority of SYM metabolites peaked 2 h prior to dawn (04:00 h), we hypothesize that an accumulation of these compounds in the hemolymph may reflect the prolonged activity of the light organ symbionts throughout the night.

Within the adult light organ, daily rhythms of the symbiosis that have been previously described (Wier et al., 2010; Schwartzman et al., 2015) appear to be linked to the patterns of daily metabolite abundance in the hemolymph described here. One such rhythm is the pre-dawn effacement of the apical surface of the crypt epithelium, which releases membrane lipids that are used by *V. fischeri*. For example, the membrane lipid constituent myristic acid, metabolized by *V. fischeri* in the light organ (Wier et al., 2010), is highest in the hemolymph at 04:00 h, the time point nearest the dawn effacement; subsequently, myristic acid levels in the hemolymph decrease throughout the day, presumably as the symbiont population regrows and metabolizes such host-derived fatty acids from degraded lipids. Of the other five lipids significantly affected by colonization, four (80%) similarly exhibited their lowest abundance in SYM hemolymph at 19:00 h (Fig. 3).

In the mature symbiosis, the appearance of *N*-acetylglucosamine (GlcNAc) in the light organ drives *V. fischeri* to shift from anaerobic respiration of glycerol during the day to fermentation of GlcNAc at night (Wier et al., 2010). While not statistically different when all time points are compared between SYM and APO animals, glycerol levels in the hemolymph exhibited a shift in abundance in SYM animals similar to pattern #1 (Fig. 3), with the lowest abundance occurring at 11:00 h and highest at 04:00 h. This rhythm is consistent with the presence of abundant GlcNAc in the crypts during the night (19:00 h and 04:00 h) resulting in a decreased utilization of glycerol by *V. fischeri* (Pan et al., 2015; Schwartzman et al., 2015) and, subsequently, an increase in glycerol in the circulating hemolymph. GlcNAc was at its lowest abundance in the hemolymph at 19:00 h (Fig. 3). The decreased level of GlcNAc at 19:00 h is consistent with a nightly shift to GlcNAc utilization by symbionts in the light organ. The increase in GlcNAc from 19:00 h to 04:00 h is not yet understood; however, this pattern is consistent with a previous finding that the number of chitin-carrying hemocytes in the crypts peaks near dusk, before returning to a lower level prior to dawn (Schwartzman et al., 2015). Taken together, the abundances of glycerol and GlcNAc in the hemolymph reflect the patterns of symbiont catabolism within the light organ, and suggest that they may be transfer metabolites, transported from the host to the symbiont.

Finally, in female squid, the systemic effect of light organ colonization is difficult to assign because of the presence of a second symbiotic organ, the accessory nidamental gland (ANG). The metabolic activities of the ANG's symbiotic consortia are undefined, which complicates an interpretation of the source of metabolites found in the hemolymph. Nevertheless, colonization of the light organ does have a reproducible effect on the hemolymph metabolome of females (Fig. 1C), although the changes differ from those observed in males. While nearly all of the metabolites that were significantly altered by symbiosis were higher in SYM males, they were lower in SYM females. Although the majority of metabolites displayed such sexual dimorphism in their response to colonization, levels of sugar #6 were strongly increased in SYM hemolymph of both males and females. Furthermore, the levels of myo-inositol, while not significantly different after correcting for multiple comparisons, exhibited a trend for down-regulation in SYM hemolymph of both sexes (Fig. 4D). In contrast, myo-inositol is lower in gnotobiotic mice relative to conventionalized littermates in numerous studies, and has been shown to regulate osmosis and phagocytosis (Claus et al., 2008; Chen et al., 2015), suggesting a possible role in animal–bacteria interactions. A promising avenue for research on the squid–vibrio symbiosis would be to examine laboratory-reared SYM and APO females in which colonization of the ANG can be controlled.

### Conclusions

Studies using mammals have long described the numerous effects microbes can have on host metabolism. However, the sterile conditions required to raise gnotobiotic mammals eliminate any similar effects arising from non-symbiotic microbes found in the host's ambient environment. The ability to raise SYM and APO squid with the naturally occurring consortium of marine microbes still present in the animal's seawater environment provides the opportunity to define the effects of a specific symbiont species on host blood composition. By applying metabolomics to the squid–vibrio symbiosis, this study shows how a natural colonization can not only affect the symbiotic organ, but also change overall host biochemistry by influencing daily rhythms within the metabolome.
